# Mieloma múltiple IgE: detección y seguimiento

**DOI:** 10.1515/almed-2021-0063

**Published:** 2022-01-04

**Authors:** Beatriz Nafría Jiménez, Raquel Oliveros Conejero

**Affiliations:** Servicio de Análisis clínicos, Hospital Universitario Donostia, San Sebastián, País Vasco, España

**Keywords:** inmunoglobulina E, mieloma múltiple, proteinograma

## Abstract

**Objetivos:**

Aportar un nuevo caso de mieloma múltiple por inmunoglobulina E (IgE), isotipo muy infrecuente ya que representa <0,1% de todos los pacientes con esta gammapatía monoclonal. Destacar la importancia del estudio de proteínas con una correcta detección, cuantificación e identificación del componente monoclonal, así como las principales consideraciones a tener en cuenta en el laboratorio clínico para un adecuado abordaje.

**Caso clínico:**

Paciente varón de 45 años que, tras presentar dolor intenso en el codo de 5 semanas de evolución, es diagnosticado de mieloma múltiple IgE-Kappa gracias a las pruebas de laboratorio, junto con el análisis radiológico y de la médula ósea. Como tratamiento, el paciente recibe un esquema de inducción quimioterapéutico antes de someterse a un trasplante autólogo de progenitores hematopoyéticos. Actualmente continúa en seguimiento.

**Conclusiones:**

El estudio de proteínas por parte del laboratorio clínico a través del proteinograma y la inmunofijación han permitido detectar un componente monoclonal de tipo IgE-Kappa en un paciente antes de que presentara una sintomatología CRAB (hipercalcemia, afectación renal, anemia y dolor óseo) clásica asociada al mieloma múltiple, ayudando a un diagnóstico y tratamiento precoz.

## Introducción

Las gammapatías monoclonales representan un grupo heterogéneo de enfermedades caracterizadas por la proliferación clonal de linfocitos B o células plasmáticas productoras de inmunoglobulina (Ig). Dichas células secretan la molécula de Ig completa y/o fracciones como las cadenas ligeras libres (CLL), pudiéndose detectar en suero u orina como un componente monoclonal (CM) o paraproteína, también llamada proteína M. El espectro clínico es amplio e incluye enfermedades malignas clásicas, como el mieloma múltiple (MM) o la macroglobulinemia de Waldenström, trastornos relacionados con paraproteínas clonales tales como amiloidosis de cadena ligera, y la discrasia premaligna de células plasmáticas, denominada gammapatía monoclonal de significado incierto (GMSI/MGUS) [1].

Concretamente, el MM es la segunda neoplasia maligna hematológica más común en países desarrollados y representa alrededor del 1% de todos los cánceres. Cada año se diagnostican aproximadamente 40.000 casos nuevos en Europa y unos 2.200 en España, cuya edad media de aparición está en torno a los 65 años, aunque puede aparecer a partir de los 45 años [2]. En función del tipo de cadena pesada del CM, el MM puede clasificarse en: IgG (representando alrededor del 52%), IgA (21%), IgD (2%), IgM (0,5%) y excepcionalmente IgE (<0,1%). Asimismo, un 16% de los casos de MM son secretores de CLL (Kappa o Lambda) y algunos casos son no-secretores [3].

Para su estudio, la participación del facultativo especialista de laboratorio es clave, sobre todo para la detección, cuantificación e identificación del CM en suero y orina, aportando información muy importante sobre la enfermedad, su evolución y respuesta al tratamiento. Esto permitirá un correcto diagnóstico y estadificación, otorgando una adecuada evaluación pronóstica y toma de decisiones terapéuticas.

Con este trabajo se pretende aportar un nuevo caso de MM IgE que, debido a su baja frecuencia, desde su descripción inicial en 1967 [3] solo se han reportado aproximadamente 63 casos en lengua inglesa [4].

## Caso clínico

Varón de 45 años que acude al servicio de Urgencias del Hospital Universitario Donostia-San Sebastián (País Vasco, España), por presentar dolor intenso en el codo derecho mecánico desde hace 5 semanas, sin otra sintomatología acompañante. Como antecedentes personales presenta hipertensión arterial y cambios degenerativos en la columna dorsal y hombro derecho desde hace un año. Asimismo, refiere pérdida de estatura de 2 cm.

La exploración física es normal, pero ante la sospecha de epicondilitis derecha se le realiza una radiografía donde se observa una lesión lítica diafisaria. Ante estos hallazgos, el paciente permanece ingresado para completar el estudio (Tabla 1).

**Tabla 1:** Resultados de las pruebas de laboratorio al ingreso.

	Resultado	Valores de referencia (VR)
**(A) Bioquímica**		
Glucosa, mg/dL	104	70–110
Creatinina, mg/dL	0,73	0,7–1,2
Urea, mg/dL	49	10–65
Proteínas totales, g/dL	7,8	6,6–8,7
Calcio, mg/dL	9,6	8,6–10,2
β2-microglobulina, mg/L	2,3	0,8–2,2
Bilirrubina total, mg/dL	0,2	0,0–1,1
LDH, U/L	115	135–250
PCR, mg/L	9,8	0–5
Hierro, µg/dL	61	59–158
Ferritina, ng/mL	131	30–400
Transferrina, mg/dL	262	200–400
Proteínas orina de 24 horas, mg/24 h	101	28–141
**(B) Hemograma**		
Hematíes, ×10^6^/µL	4,1	4,3–5,6
Hemoglobina, g/dL	12,1	13–17
Hematocrito, %	38	40–50
VCM, fL	93,4	80–97
HCM, pg	29,7	27–33
CHCM, g/dL	31,8	32–36
ADE, %	14,8	11,5–15,6
Plaquetas, ×10^3^/µL	365	140–400
Leucocitos, ×10^3^/µL	6,37	3,8–10
Neutrófilos, ×10^3^/µL (%)	3,09 (48,5)	1,6–7,5 (40–75)
Linfocitos, ×10^3^/µL (%)	2,3 (36,1)	0,9–3,5 (19–48)
Monocitos, ×10^3^/µL (%)	0,8 (12,2)	0,2–0,9 (3,5–12)
Eosinófilos, ×10^3^/µL (%)	0,1 (2,2)	0,0–0,6 (0,5–7,0)
Basófilos, ×10^3^/µL (%)	0,1 (0,5)	0,0–0,2 (0–1,5)
Morfología de sangre periférica	Serie blanca: sin alteraciones morfológicas destacables. No se observan células plasmáticas. Serie roja: Rouleaux. Serie plaquetar: anisotrombia sin dismorfias destacables.	
**(C) Proteinograma**		
Albúmina, g/dL (%)	4,5 (58,5)	3,5–4,8 (55,8–66,1)
α1, g/dL (%)	0,3 (4,0)	0,1–0,3 (2,9–4,9)
α2, g/dL (%)	0,8 (10,7)	0,5–0,9 (7,1–11,8)
β, g/dL (%)	0,6 (8,3)	0,6–1,1 (8,4–13,1)
γ, g/dL (%)	1,4 (18,5)	0,7–1,6 (11,1–18,8)
CM, g/dL (%)	1,33 (17,2)	–
**(D) Inmunoglobulinas y cadenas ligeras libres en suero**		
IgG, mg/dL	333	700–1.600
IgA, mg/dL	14	70–400
IgM, mg/dL	6	40–230
IgE, kUA/L	4**.**540**.**000 (1,1 g/dL)	0,0–114,0
CLL κ, mg/L	222,4	3,3–19,4
CLL λ, mg/L	1,9	5,7–26,3
Ratio K/λ	117,69	0,26–1,65

(A) Bioquímica sérica (COBAS c702 Roche^®^, Barcelona, España). (B) Hemograma (SYSMEX XN-9100 Roche^®^, Barcelona, España) y morfología de sangre periférica (Cellavision^®^). (C) Proteinograma sérico (Capillarys-2 Sebia^®^, Barcelona, España). (D) Cuantificación de inmunoglobulinas (COBAS c702 Roche^®^) y cuantificación de cadenas ligeras libres en suero (Freelite Assay, The Binding Site^®^, Barcelona, España). LDH, lactato deshidrogenasa; PCR, proteína C reactiva; VCM, volumen corpuscular medio; HCM, hemoglobina corpuscular media eritrocitaria; CHCM, concentración de hemoglobina corpuscular media; ADE, amplitud de distribución eritrocitaria; CM, componente monoclonal; CLL, cadenas ligeras libres.

Los resultados de la analítica sérica mostraron como datos significativos un aumento de β2-microglobulina y una ligera anemia con presencia de Rouleaux en la serie roja (Tabla 1A, B).

**Figura 1:**
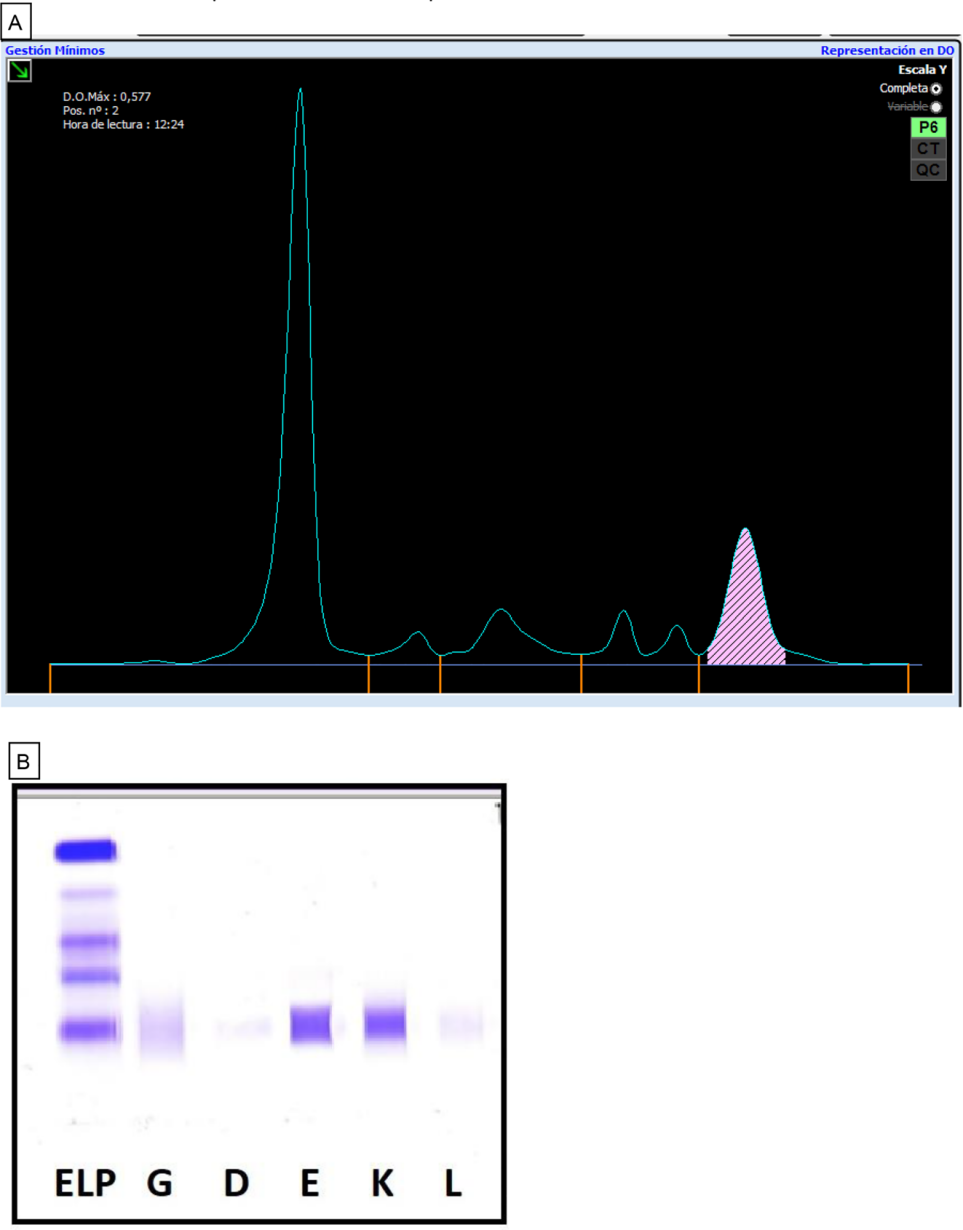
Estudio de proteínas séricas del paciente. (A) Proteinograma en suero (Capillarys-2^®^ Sebia), donde se señala un pico correspondiente al CM sobre la fracción γ. (B) Inmunofijación en suero (Hydragel IF, Hidrasys Sebia^®^), donde se identifica el CM de tipo IgE-Kappa (bandas en los carriles correspondientes a los antisueros anti-igE y anti-CLL Kappa).

En el proteinograma realizado en suero por electroforesis capilar se observa un pico monoclonal en la fracción gamma (γ) que es cuantificado como 1,33 g/dL (Figura 1A). Mediante inmunosustracción usando antisueros anti-IgG, IgA, IgM, Kappa y Lambda, este pico fue tipificado como CM de CLL-Kappa, resultado que debe confirmarse y completarse mediante inmunofijación incluyendo los antisueros específicos anti-IgD y anti-IgE. Esta inmunofijación exhibió una banda en el carril para IgE y otra banda para la cadena ligera Kappa confirmando la presencia de una proteína monoclonal IgE-Kappa (Figura 1B).

En cuanto a la cuantificación de inmunoglobulinas séricas, destaca la elevada concentración de IgE (ImmunoCAP, Phadia^®^, Barcelona, España), y la ratio de cadenas ligeras libres κ/λ en suero que fue de 117,69 (Tabla 1D). En el análisis de orina de 24 horas, el paciente no muestra ni proteinuria ni se observa CM Bence-Jones mediante electroforesis capilar e inmunofijación.

A la vista de estos resultados, se decide estudiar la médula ósea y se objetiva una infiltración del 30% de células plasmáticas donde la práctica totalidad presenta un inmunofenotipo aberrante por citometría de flujo (CD138+/CD38+low/CD19-/CD45+het/CD56++/CD117-/CD27+het/CD81+het). El análisis citogenético mediante hibridación fluorescente *in situ* (FISH) de células plasmáticas purificadas fue normal, siendo el reordenamiento IGH-CCND1, t(11;14) (q13;q32) la única anomalía detectada.

Ante todos estos hallazgos, de acuerdo con los criterios diagnósticos del Grupo de Trabajo Internacional del Mieloma (IMGW) [1, 5], el paciente es diagnosticado de Mieloma Múltiple IgE-Kappa, estadio Durie-Salmon IIIA, ISS-R:1 [1].

## Discusión

El MM IgE es el tipo menos frecuente de esta gammapatía monoclonal y, como consecuencia de ello, el conocimiento de esta condición se recopila de informes aislados y algunas pequeñas series de casos [3, 6].

Las células plasmáticas neoplásicas y la producción de Ig monoclonal provocan daño en órganos y tejidos, dando como resultado las características clínicas típicas del MM que incluyen hipercalcemia, daño renal, alteración en la hematopoyesis con anemia y lesiones óseas osteolíticas; recogidas bajo el acrónimo CRAB [7]. Esta sintomatología también está presente en los casos de MM IgE reportados y diferentes estudios sugieren que los resultados de laboratorio son similares a los de isotipos más frecuentes de MM [8]. No obstante, el caso presentado no cumple parte de estos criterios CRAB clásicos, puesto que los niveles de calcio séricos y la función renal eran normales, por lo que la detección del CM en suero y su identificación como IgE-Kappa mediante inmunofijación fue clave para su diagnóstico, junto con otras pruebas como la radiografía y el estudio de médula ósea. Por tanto, una prueba tan asequible como el proteinograma y la cuantificación e identificación de la Ig monoclonal son fundamentales para establecer un diagnóstico precoz y en la toma de decisiones terapéuticas, afectando a la supervivencia de los pacientes. Por ello, en línea con otros autores y el IMGW, para el cribado del CM en el laboratorio clínico se recomienda seguir un protocolo de estudio que incluye la electroforesis, la determinación de las CLL en suero y la inmunofijación, aumentando la sensibilidad y, por lo tanto, la capacidad de detección [9].

De los datos conocidos de MM IgE, incluido el paciente actual, se ha obtenido una distribución por sexo y edades de inicio con tasas hombre:mujer 20:18 y entre 38–80 años [3, 6].

En cuanto al tratamiento de primera línea en pacientes con MM relativamente jóvenes (<70 años), se recomienda un tratamiento de inducción para la recolección de las células progenitoras hematopoyéticas, seguido de un acondicionamiento con quimioterapia mieloablativa para eliminar las células anómalas del organismo, generar espacio en la médula ósea para las nuevas células y evitar el rechazo de las mismas por parte del receptor. Posteriormente, se realiza el trasplante autólogo de los progenitores hematopoyéticos de sangre periférica (TASPE) [7].

**Figura 2:**
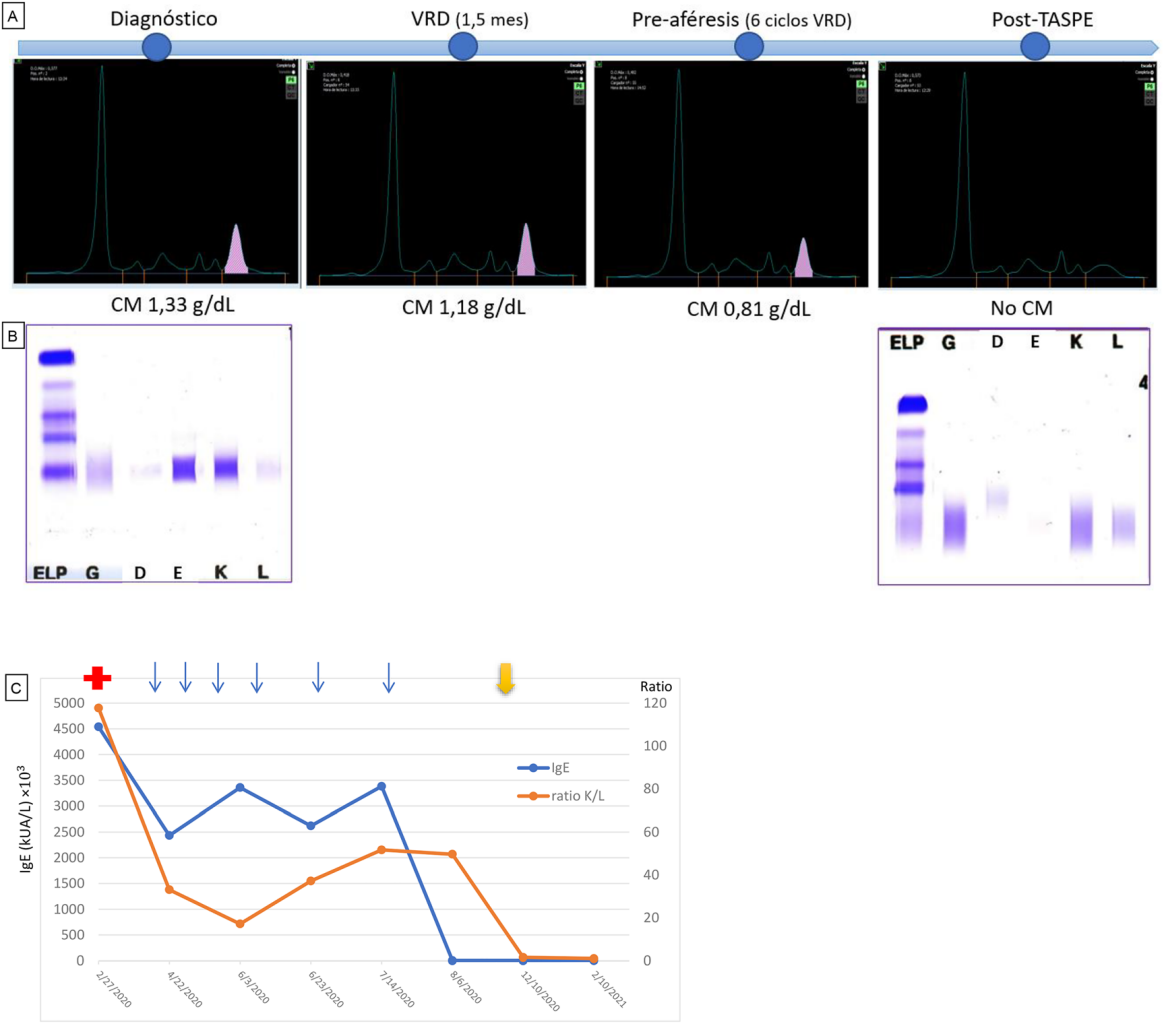
Evolución del estudio de proteínas del paciente. (A) Evolución del proteinograma sérico: al diagnóstico, con el tratamiento VRD, pre-aféresis y post-TASPE. Se observa que el pico monoclonal sobre la fracción γ va disminuyendo. (B) Geles de inmunofijación sérica. Se observa el CM de tipo IgE-Kappa al diagnóstico y su desaparición tras el trasplante. (C) Representación de la evolución de los niveles de IgE séricos y el ratio Kappa/Lambda. La cruz roja inicial indica el diagnóstico de la patología, cada flecha azul representa un ciclo de VRD y la flecha amarilla final indica la realización del TASPE. Se observa una disminución de la concentración en suero de IgE y de CLL-Kappa.

En la entidad por IgE, las estrategias terapéuticas generalmente empleadas son las mismas que las de los tipos más comunes de MM ya que se han reportado resultados similares [10, 11]. En nuestro caso, el paciente ha recibido un esquema de inducción de 6 ciclos con el triplete VRD que incluye Bortezomib (inhibidor delproteasoma 26S), Lenalidomida (inmunomodulador) y Dexametasona (glucocorticoide); y se le ha realizado un TASPE. Actualmente, ha logrado una respuesta completa con enfermedad mínima residual positiva (persistencia de una cantidad muy pequeña de células malignas tras el tratamiento), de acuerdo con los criterios del IMGW [7], y continúa en seguimiento. En el último análisis (1 año después del diagnóstico) no se observa CM IgE-Kappa ni en el proteinograma ni en la inmunofijación en suero (Figura 2A, B). Asimismo, los niveles de IgE y la ratio de caderas ligeras Kappa/Lambda han ido disminuyendo con el tratamiento (Figura 2C).

Cabe señalar que a la hora de cuantificar la IgE sérica debe considerarse el efecto prozona [12]. Esta limitación del inmunoensayo es debido a que el método está basado en la reacción antígeno-anticuerpo en una mezcla equilibrada para la detección. De manera que, si existe un exceso de concentración de IgE en la muestra del paciente, se va a producir la saturación del anticuerpo anti-IgE, quedando parte del analito IgE sin unir y sin formar los complejos correspondientes perdiendo su detección. Esto puede derivar en un resultado incorrecto mientras que una validación conjunta de todas las pruebas puede prevenirlo [13]. En nuestro caso, la concentración de IgE al diagnóstico fue de 4**.**540**.**000 kUA/L (1,1 g/dL) siendo muy similar a la calculada en el proteinograma (1,33 g/dL).

En cuanto a las anomalías citogenéticas (FISH), sólo se detectó el reordenamiento IGH-CCND1 t(11;14)(q13;q32) sin otras alteraciones cromosómicas. En este sentido, mencionar que la translocación t(11;14) se considera un sello distintivo del MM IgE, IgM y MM no-secretor, pues dicha traslocación presenta una frecuencia significativamente mayor en estos subtipos raros de MM [14].

Respecto al pronóstico de estos pacientes, anteriormente se ha postulado una menor supervivencia media (12,5 meses) incluso después del TASPE, así como una mayor tasa de proliferación a leucemia de células plasmáticas [8]. No obstante, muchos de estos datos proceden de épocas anteriores a los nuevos fármacos inmunomoduladores e inhibidores del proteasoma; mientras que la evidencia de informes publicados más recientemente sugiere que los resultados en pacientes tratados con estos nuevos agentes son notablemente mejores [10, 11]. Sin embargo, el mayor retraso diagnóstico en estos casos aislados puede favorecer un curso clínico más agresivo. Por tanto, la teoría anteriormente aceptada de que el MM IgE cursa con peor pronóstico se está sometiendo a debate e incluso se ha reportado un caso que sobrevivió más de 20 años, finalmente falleciendo de comorbilidades crónicas con 77 años [3].

En conclusión, el MM IgE es un tipo poco común de mieloma cuyas características clínicas y esquemas de tratamiento son similares a las de los otros isotipos. Aunque se necesitan más estudios para poder establecer conclusiones generales sobre esta entidad y que nuestros resultados deben interpretarse con cautela, queremos señalar que este caso amplía el conocimiento sobre la detección de un CM de tipo IgE.

## Cinco puntos clave

– El mieloma múltiple por inmunoglobulina E es un trastorno poco frecuente, pero debe considerarse este isotipo ante la identificación inicial de un componente monoclonal.
– El empleo correcto de las técnicas de estudio de proteínas permite un adecuado diagnóstico y estadificación, siendo fundamentales para dar una correcta información pronóstica al paciente y poder evaluarlo.
– La detección del componente monoclonal se recomienda mediante un proteinograma por electroforesis. Asimismo, el método de referencia para su identificación es la inmunofijación con antisueros específicos anti-IgE.
– El efecto prozona es un fenómeno de laboratorio conocido que puede complicar la interpretación de ensayos cuantitativos dando resultados falsamente normales. Esto es especialmente importante en los casos de MM IgE que, por ser infrecuentes, pueden infravalorarse.
– Establecer unos protocolos de detección, identificación de isotipo y seguimiento de gammapatías monoclonales mediante el uso de electroforesis, inmunofijación, cuantificación de inmunoglobulinas y de cadenas ligeras libres, permite comprender la importancia de una detección precoz y su tratamiento en la evolución de la enfermedad.
